# Supplementary material for: A Pragmatic Bilayer Selective Emitter for Efficient Radiative Cooling under Direct Sunlight
Source: Materials (Basel). 2019 Apr 12;12(8):1208. doi: 10.3390/ma12081208 (PMC6514789; doi:10.3390/ma12081208)
Supplement: Supplementary file 1 [file materials-12-01208-s001.pdf]

materials

Article

A Pragmatic Bilayer Selective Emitter for Efficient Radiative Cooling under Direct Sunlight

Yiwei Liu [1,2,3], Anqi Bai [1,2,3], Zhenggang Fang [1,2,3,*], Yaru Ni [1,2,3,4,*], Chunhua Lu [1,2,3,*] and Zhongzi Xu [1,2,3]

1. State Key Laboratory of Materials-Oriented Chemical Engineering, College of Materials Science and Engineering, Nanjing Tech University, Nanjing 210009, China; 2452615070@njtech.edu.cn (Y.L.); aqbai@njtech.edu.cn (A.B.); zzxu@njtech.edu.cn (Z.X.)
2. Jiangsu Collaborative Innovation Center for Advanced Inorganic Function Composites, Nanjing Tech University, Nanjing 210009, China
3. Jiangsu National Synergetic Innovation Center for Advanced Materials (SICAM), Nanjing Tech University, Nanjing 210009, China
4. Key Laboratory of MEMS of Ministry of Education, Southeast University, Nanjing 210096, China

* Correspondence: zgfang@njtech.edu.cn (Z.F.); nyr@njtech.edu.cn (Y.N.); chhlu@njtech.edu.cn (C.L.)

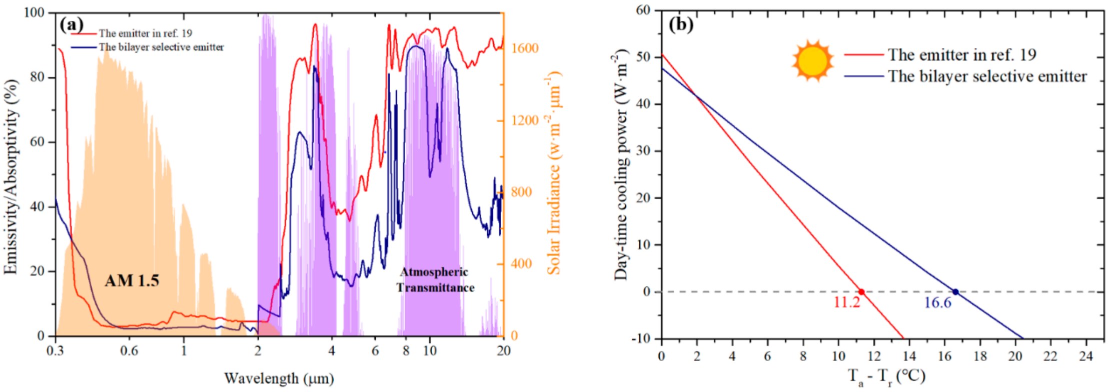

Figure S1. the comparsion of (**a**) the solar and IR emissivity/absorptivity and (**b**) the day-time cooling power between between the bilayer selective emitter and a selective emitter in ref. [19].
